# Impact of a behaviour change communication programme on net durability in eastern Uganda

**DOI:** 10.1186/s12936-015-0899-5

**Published:** 2015-09-22

**Authors:** Michelle H. Helinski, Geoffrey Namara, Hannah Koenker, Albert Kilian, Gabrielle Hunter, Angela Acosta, Leah Scandurra, Richmond Ato Selby, Kenneth Mulondo, Megan Fotheringham, Matthew Lynch

**Affiliations:** Malaria Consortium, Kampala, Uganda; Johns Hopkins University Center for Communication Programs, Baltimore, MD USA; Tropical Health LLP, Montagut, Spain; Malaria Consortium, London, UK; Johns Hopkins University Center for Communication Programs, Nairobi, Kenya; Johns Hopkins University Center for Communication Programs, Kampala, Uganda; John Snow International, Kampala, Uganda; United States Agency for International Development, Washington DC, USA

**Keywords:** LLIN, Malaria, ITN, Care and repair, Durability, BCC, Uganda

## Abstract

**Background:**

The importance of net durability and the average useful life of a net is increasingly recognized as one of the critical factors that determine how often nets need to be replaced. A study to assess the effect of a net care and repair behaviour change communication (BCC) programme on net durability was conducted in one district in Eastern Uganda with a district in a neighbouring region serving as a comparison. Both districts had received LLINs in September of 2012.

**Methods:**

The intervention was comprised of radio programmes, school and community events. Two-stage cluster sampling household surveys to assess net condition, exposure to BCC messages, and attitudes towards net care and repair were conducted in both districts at baseline (2–3 months post net distribution) and endline (20–21 months post distribution). Net condition was assessed using the proportionate hole index, with nets being classified as either serviceable or too torn.

**Results:**

The intervention led to an additional 31.2 % increased exposure to net care and repair messages in the intervention district. Respondents in the intervention district had a more positive attitude towards net care and repair (32 % of respondents were classified as having a very positive attitude compared to 10 % in the comparison district), which was positively associated with the number of channels through which messages had been received (P < 0.001). Nets belonging to respondents with a very positive attitude were more often categorized as serviceable (80.2 %) compared to respondents with a poor/average attitude (66.4 %; odds ratio: 2.05, P = 0.028); however, this was only observed for the net brand with the greater physical integrity. Additionally, socio-economic status was a significant predictor of net condition. Although nets in the intervention district had significantly more repairs done per net, the act of repairing alone did not improve net condition.

**Conclusions:**

In conclusion, the evaluation showed that the BCC programme resulted in improved knowledge and attitudes towards care and repair, which impacted positively on net condition. Repairs alone were not sufficient to improve net condition. Additional research on which care behaviours and attitudes are most associated with improved net condition would help BCC planners hone their campaigns.

## Background

Malaria prevention with long-lasting insecticidal mosquito nets (LLIN) has seen a tremendous scale-up in sub-Saharan Africa in recent years [1]. As many countries have now achieved high ownership coverage with LLINs and are approaching the universal coverage target of one net for every two people of the population at risk as recommended by the World Health Organization (WHO) [1], the question of how these high coverage levels can be maintained becomes the focus of discussion. In this context, the importance of net durability and the “average useful life” of a net is increasingly recognized as one of the critical factors that determine the frequency at which nets need to be replaced, and methods to measure net durability in the field have been established [2]. There is a paucity of data available on how the durability of a net is influenced by behaviour of net maintenance, care and repair and whether behavioural change communication (BCC) interventions could improve net life-span.

Nets are exposed to a number of factors that may impact on its durability. Rodents have been implicated as a major cause of net damage in some communities [3], but also children, fire, sharp objects, and certain sleeping materials such as mats, are mentioned by owners of bed nets as common sources of damage [4–6]. Such damages may be partly associated with net hanging and storing habits, as well as other household practices around food storage and preparation which could attract rodents. Besides mechanical damage, washing and drying practices of nets impact on the insecticidal properties of the net, and there is some evidence that over-washing may also deteriorate fabric integrity [5, 7]. While it is unclear to what extend household owners can prevent some of these damages, it is likely that increased knowledge of net care and awareness of potential net damage are beneficial to improve the overall lifespan of the net.

Besides net care, repair of nets that have acquired holes is advocated to maintain an effective barrier against the mosquito vector and to increase overall lifespan of the net. In this light, the early repair of small holes, before they become bigger, has been put forward as an effective way to repair nets [6]. Where investigated, net repair behaviour can vary considerably between regions, with nets in some communities showing few signs of repair [5, 8] while in others more repairs are observed [9, 10]. The effect of such repair behaviours on net longevity has not been studied to a great extent, yet in Kenya nets that showed signs of repair did not have an improved overall net condition compared to unrepaired nets [5]. Authors noted, however, that repairing of nets was not a common practice in this study site, and that nets with signs of repair had many holes [5].

Intensive BCC programmes have been associated with increases in LLIN use in Cameroon [11] and Zambia [12], and with increases in other malaria preventive behaviours elsewhere in sub-Saharan Africa [13–16]. However, only two BCC interventions focusing on care and repair are found in the literature. Implemented in the Gambia, a 2006 study observed a significant increase in repair behaviours, specifically the proportion of holes repaired, following an intervention composed of community song competitions and posters, but did not measure overall durability [17]. A recent study in Nigeria, similar to the present one in design and scope, is described elsewhere [18].

The main goal of this study was to determine how the durability of a net is influenced by behaviours of net maintenance, care and repair and whether BCC could significantly impact on net durability.

## Methods

### Study areas

The study took place in Eastern Uganda. Serere district was selected as the intervention district receiving the BCC programme while Kaliro, a nearby district, was used as the comparison district. The two districts are geographically and culturally similar with similar housing, however different languages are spoken (Teso in Serere and Lusoga in Kaliro) and districts do not share borders.

The Government of Uganda distributed 22.2 million LLINs as part of a national universal coverage campaign from 2012 to 2014. The intervention and comparison district participated in the four-district pilot phase of the universal coverage campaign (UCC) and received their nets in September 2012.

### Design of the BCC intervention programme

**Fig. 1:**
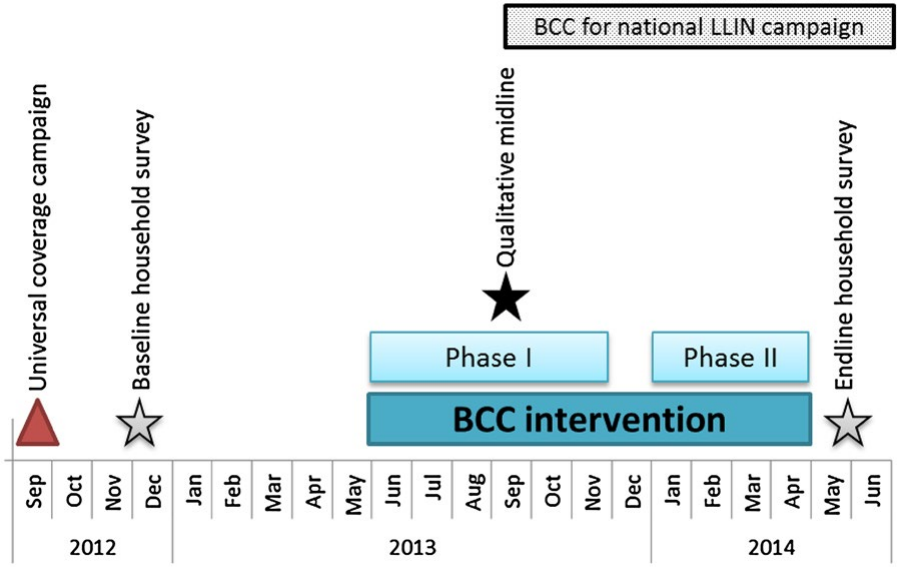
Timing of universal coverage campaign, BCC intervention, and surveys. In addition, the time period when BCC activities as part of the national LLIN campaign took place is indicated

The net care and repair BCC programme started in the intervention district in June 2013 (9 months post-distribution) and ran for 10 months until April 2014. The campaign was designed by BCC experts jointly with district leaders, school teachers and community health workers. Activities were conducted in two phases (June–November 2013 and January–April 2014; Fig. 1). The two-phase approach was used to give planners an opportunity to refresh campaign approaches, prevent audience fatigue and incorporate monitoring and midline qualitative data. Phase I was based on data from an unpublished qualitative study on the culture of net use in Uganda; while phase II used data from a qualitative midline assessment [19] and monitoring reports from phase I. Target audiences for the interventions were mainly mothers and primary school children in classes P2-P7 (ages 9–15). Both qualitative studies had identified women as the main household members responsible for net care and repair and children’s behaviour as a leading cause of damage. Men were not a primary audience of the campaign but field coordinators tried to encourage and increase male participation. During phase I, community activities were mostly attended by women and children. However, during phase II, more men attended the events although overall women were still predominant.

The key messages of the campaign focused on benefits of net care, methods to take care of and repair nets including early repair of holes and tying up the net and net washing practices. The messages were selected based on the theory that perceptions of malaria risk, the feasibility of net care and repair, and the value of repaired nets are vital to behaviour change, as described in the conceptual model by Koenker et al. [18]. The activities that were organized were mass media and community mobilization events. As part of the mass media activities, 3230 radio spots, 2188 DJ mentions (i.e., short 45-s scripts) and 19 radio talk shows were aired on two radio stations in the intervention district. In addition posters were used in health facilities, market areas and schools of the 30 villages in which the household surveys took place.

Community mobilization events took place in the 30 villages and their associated schools and consisted of a number of activities. Community events included compound dialogues, 34 song competition events, home visits, and 45 forum theatre performances, and were mainly led by village health team (VHT) members. District education leaders set aside two arts and craft class sessions for net care and repair and created a curriculum on this topic. School events consisted of net care and repair classes, quizzes, debates, and music, dance and drama competitions, and were mainly organized by teachers. The radio shows featured community and school events and their participants and brought district leaders and project members to discuss net care and repair. Radio and print materials and drama scripts were pretested with groups of men and women to ensure the acceptability and relevance of the messages.

The project paid for the cost of radio spots, DJ mentions, and talk shows, the training of teachers and VHTs, supervision visits and public address systems and awards for selected community activities. VHTs, troupes and teachers were not compensated for their time but they received allowances for food and transportation for trainings, the semi-final and final rounds for the song competition, the school music, dance and drama competition and for all forum theater events. VHTs and teachers were also equipped with kits for demonstrating net care and repair that were used in the school, community and drama activities.

Uganda distributed LLINs throughout the country as part of the main national campaign from September 2013 onwards. BCC messaging to support this campaign was ongoing through national and regional channels (mainly radio) to inform people about the distribution and how to use and take care of their nets, and it is likely that respondents in both districts were exposed to some degree to these messages.

### Surveys

The principal evaluation design was that of repeated, cross-sectional, two-stage cluster sampling household survey representative of the rural population of the two selected districts participating in the LLIN distribution. Data was collected at baseline (2–3 months post-net distribution) and at endline (18 months post-baseline, and 20–21 months post-distribution; Fig. 1). Qualitative data was collected at midline to monitor the reach and reception to the campaign and collect further insights on behavioural motivators and barriers that could be used to improve the programme and its methods and results are described elsewhere [19].

A sample of 30 clusters with 15 households each (450 households) per site and time point was used. First stage sampling involved selection of clusters using probability proportionate to size (PPS). Clusters were taken as villages, and the number of households in a village was obtained from LLIN campaign registration lists. At second stage, households were randomly selected from a listing of all households in the village. Clusters for the household survey were selected at baseline and maintained during endline while households were randomly selected at each survey round.

Households were eligible if they had received nets as part of the September 2012 campaign. Survey respondents were at least 18 years of age. Where possible, these respondents were the head of household or his/her spouse. Prior to field survey activities, local authorities and chiefs were informed of the purpose and expected time of the survey and their support sought. Communities were sensitized and mobilized in order to obtain maximum cooperation for the survey.

The household questionnaire asked questions regarding household characterization (composition, assets etc.), net care and repair behaviour of all nets in household, including exposure to care and repair messages, nets received from campaign, loss and reasons for loss, use of nets, and care and repair behaviour of campaign nets, including net hole assessment. The endline questionnaire contained a number of additional questions not asked during baseline to provide more detailed information to assess the BCC programme. For these questions only endline comparisons between intervention and comparison district are presented.

### Oversampling Olyset nets at endline

During the baseline survey it was found that while the comparison district had exclusively received the LLIN Olyset^®^ made from polyethylene, the intervention district had primarily received the LLIN PermaNet^®^ made from polyester with only a small number of Olyset nets distributed in a few clusters. To account for this unanticipated and uneven distribution, during the endline survey Olyset nets were purposively oversampled in the three clusters where the baseline survey had identified them alongside PermaNet. In these clusters, following the random sample as above, all households were visited and interviewed if Olyset nets were present. In two of these clusters, a nearby village was also sampled to increase sample size. The full questionnaire was administered to the respondents yet questionnaires were marked to distinguish them from the random sampling ones.

### Outcome measures

Each campaign net found in the household was assessed for physical condition and signs of repair after permission was given by the respondent. All sides and roof of the net were examined for holes or repairs, with existing holes counted and categorized into four different sizes (size 1: 0.5–2 cm, size 2: 2–10 cm, size 3: 10–25 cm and size 4: larger than 25 cm in diameter) based on WHO guidelines [20]. Nets that had too many holes to count, or were damaged severely such that large parts of the nets were missing, were scored as having 10 size 4 holes. The presence and number of repaired holes was noted but these were not counted as holes. Partially repaired holes were counted as holes and as partial repairs. Data from the net hole assessment was transformed into the proportionate Hole Index (pHI) for each net [20]. Based on the pHI, each net was categorized as “good”, “damaged” or “too torn” condition [21]. Any net that is not “too torn” is considered “serviceable”.

Net attrition was calculated for the campaign nets received and defined as the proportion of originally received nets (as reported by the households) which had been (1) lost overall (overall attrition) and for (2) those nets which had been lost due to wear and tear (thrown away, destroyed) at the time of assessment (attrition due to wear and tear). Net survival was then defined as the proportion of LLINs functionally surviving to time x, which is calculated as the proportion of received nets not given away for use by others that are still present and in “serviceable” condition. Nets received but given away for use by others or stolen were excluded from the denominator for the net survival.

### Data processing and analyses

A double data entry and validation system was designed in EpiData 3.1 software. Analyses were conducted using Stata v12.0 (StataCorp, USA). Despite the sampling expected to generate a self-weighting sample, weighted analyses were conducted since the cluster sizes were found to have varying sizes. Sampling weights were computed and used through use of the “svy” command in Stata.

The socioeconomic status (SES) was calculated at the household level based on household characteristics and assets using a principal component analysis (PCA) and a wealth index was created from the first component. Households were then classified into wealth quintiles (five) according to their index value.

To evaluate the impact of the BCC programme, comparisons were made between districts for each survey round (baseline or endline), or between survey rounds for each district, dependent on what was most relevant. Tests of differences were analysed using Chi square tests for categorical variables and use of Student’s t-tests for continuous variables. For some indicators, a difference in differences (DID) was calculated, using either logistic regression or the “diff” command in Stata. The DID calculates the effect of the intervention on an outcome indicator by comparing difference between the change in the outcome indicator for the intervention group to the change for the comparison group.

A series of 11 positive and negative statements on net care and repair (i.e., benefits of net care and repair, ease of net care and repair) were read to respondents and their attitudes were captured on a Likert-type scale [i.e., strongly disagree (−2), somewhat disagree (−1), somewhat agree (1), strongly agree (2)] during the endline survey. Negative statements scores were inverted during analyses. A composite index was created using these responses to ascertain attitudes of respondents. Average scores were generated per respondent and were classified in three groups; poor/average attitude (<1), positive attitude (1–1.5), and very positive attitude (≥1.5). Ordered logistic regression was used to determine the association between attitude and exposure dose and attitude and number of messages heard.

Multivariate logistic regressions were generated for each LLIN brand (PermaNet and Olyset) separately using the physical net condition as the outcome variable (0 “too torn”, 1 “serviceable”) and a number of predictors were included as independent variables. Potential predictors included in the model were those known based on previous research, those independently associated with net condition, as well as others that the study team believed would impact on net condition. First, a crude model was built by assessing the relationship between each predictor on net outcome. The final adjusted model was arrived at by iterative removal of predictors whose removal had no impact on the model as determined by the Wald test, starting from least significant predictor.

### Purposively sampled households

Purposively selected households were not included in the analyses with the exception of results around net condition. Sampling weights were re-calculated and applied when the purposively sampled households were included in the analyses. Due to observed significant differences in net durability between the two net brands, for the endline dataset Olyset nets from the randomly selected households in the comparison district were compared versus the randomly and purposively sampled Olyset nets in the intervention district. In addition, a separate analysis was done for nets identified as PermaNet in the intervention district.

To ascertain that key variables were similar between random and purposively sampled households in the intervention district, data analyses were also done comparing the two groups. Besides some minor differences in distribution of answers in multiple answer questions, there were no differences between random and purposively sampled households when looking at key variables.

### Ethical clearance

Ethical clearance was obtained from the institutional review boards of the Vector Comparison Division of the Uganda Ministry of Health and the Uganda National Council for Science and Technology (SS-2982) and Johns Hopkins School of Public Health (IRB #4534 and #5549). In addition, oral consent from the participants was obtained prior to the start of the interview.

## Results

Response rates during baseline and endline surveys varied between 94 and 100 % (i.e., 425–450 households). In the intervention district, an additional 266 households were purposively sampled during the endline survey to obtain a sufficient sample of Olyset nets. This was short of the desired 425 due to limited number of clusters in which Olyset had been found at baseline and the total number of households available in these clusters.

### Household characterization

Respondents were mainly female and the head of the household or the spouse in both districts. Age of respondents was similar between districts and was on average 39.9 years (P = 0.708). Significant differences were observed in the education level of the respondents, these were overall higher in the comparison district. In contrast, reading abilities were significantly better in the intervention district, where 63.4 % of respondents were able to read to some degree compared to 51.4 % in the comparison district (P < 0.001). The percent of households that had children in school classes P2–P7 (approximately 9–15 years of age) was similar between districts and was on average 71.9 % of households.

Radio ownership was similarly high for both districts and at endline ownership was on average 71.8 % (P = 0.307). During baseline it was observed that the comparison district had significantly more households belonging to higher wealth quintiles than the intervention district (P < 0.001). Therefore, wealth quintiles were created separately for each district to determine if there was a change in distribution from baseline to endline. In the comparison district, no differences were observed in SES between endline and baseline surveys. In the intervention district, more households during endline were classified into higher wealth quintiles compared to baseline figures (P = 0.027).

Food was stored regularly in sleeping rooms in both districts (69.8 %; P = 0.317). In the intervention district, cooking occurred in rooms also used for sleeping significantly more often, although this happened only in 10 % of households (P = 0.004). Presence of rodents were reported in the large majority of households in both districts, more so in the comparison district (95.7 %) compared to the intervention district (90.2 %; P = 0.009).

Household net ownership in both districts was high, and almost all households (>98 %) owned at least one mosquito net (i.e., previously owned nets and campaign nets combined) at endline. This reflects the eligibility criteria that only included households that had received nets as part of the September 2012 campaign. Households in the intervention district owned significantly more nets (average 3.2 nets) at endline than the comparison district (average 2.7 nets; P = 0.003). At baseline, 72.4 and 77.6 % of households owned enough nets to cover all its residents assuming one net for every two people, in the comparison and intervention district, respectively. Eighteen months later, the percent of households with at least one net for every two people had dropped to 39.2 and 46.0 %, respectively, translating to a similar percent point drop in both districts of around 32 %.

### Exposure to net care and repair messages

**Fig. 2:**
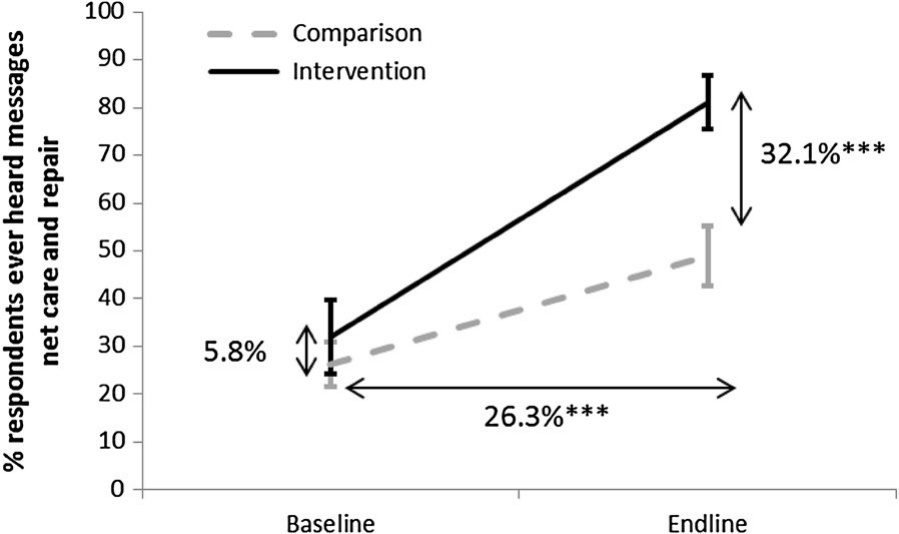
Percent of respondents [95 % CI] that ever heard messages on net care and repair, by survey and district. P values for the differences at baseline and endline between districts are indicated. Additionally, the difference of difference is indicated; where ***P < 0.001

Before the start of the BCC programme, there was no significant difference between the districts in the percent of respondents who had ever heard any messages on net care and repair, on average 29.8 %. At endline, a significantly greater percent of respondents had heard net care and repair messages compared to the baseline in both districts, yet the increase was much greater in the intervention district where at endline 81.1 % of respondents had ever heard such messages compared to 49.0 % in the comparison district (Fig. 2).

Radio was the main medium through which respondents had heard these messages at endline in both districts (81.4 %), followed by health workers (Table 1). Respondents in the intervention district had also heard messages at religious gatherings due to the tendency of VHTs to schedule their events around such venues. Other channels through which the BCC programme operated, i.e., students, community events, and posters/banners, were another source of information in the intervention district, albeit at lower recall frequency (<21 %). The exposure dose, i.e., the number of channels mentioned by respondents who had heard such messages, was similar at baseline between districts and was on average 1.5 channels (P = 0.118). However, at endline the exposure dose was significantly higher in the intervention district with 3.2 channels mentioned per respondent compared to 1.7 for respondents in the comparison district (P < 0.001). There was no significant difference in frequency of messages heard on net care and repair in both the comparison and intervention district, and the majority of people (58.0 and 66.5 % in the comparison and intervention district, respectively) had heard such messages six or more times in the past 12 months (P = 0.373).

**Table 1:** Exposure to messages on net care and repair; percent distribution of level of exposure to messages at endline

	Comparison	Intervention	P value^π^
	N = 445	N = 425	
Source of messages^a^			
Radio	82.2	81.1	0.780
Television	3.2	2.1	0.499
Village health worker	38.2	51.3	0.043
Health (facility) worker	23.1	36.8	0.051
Community leader	7.4	34.2	<0.001
Church or mosque	1.0	42.5	0.001
Family or friend	7.6	19.8	0.004
Poster or banner	0.0	14.2	0.022
Students	0.0	14.3	0.007
Teacher	0.5	2.9	0.065
Community event	5.5	20.6	0.001
Messages most remembered^a^			
Handle net carefully	57.9	62.1	0.538
Keep net away from fire/flame	8.7	18.6	0.010
Repair holes early	36.6	75.3	<0.001
Frequency of washing	24.3	12.9	0.011
Avoid bleach for washing	29.2	55.5	<0.001
Dry net in shade	36.0	55.9	0.003
Take care net prevent malaria	26.5	35.9	0.093
Inspect net for holes	4.3	17.3	<0.001
Keep net away from rats	14.8	15.7	0.833
Take care net to make last longer	15.7	17.5	0.653
Roll up/tie up when not in use	11.8	39.5	<0.001
Silent nights, Happy days: care, repair, protect	1.1	25.1	<0.001
LLIN national campaign (“Protect yourself from malaria, use a net every day”)	16.2	18.9	0.567
Other	19.3	9.3	0.031

^π^P values are shown for endline comparisons between district

^a^Only among those who heard messages on net care and repair

The main message recalled in the intervention district was to “repair holes early” (Table 1), followed by “handle the net carefully”. This latter message was also recalled in the comparison district at a high rate. A message specific to the BCC programme (Silent nights, Happy days: care, repair, protect) was remembered by one quarter of respondents in the intervention district, against only 1.1 % of respondents in the comparison district. Respondents from the intervention district were also more likely to remember messages around tying up a net when not in use, inspecting nets for holes, drying nets in the shade, avoiding bleach for washing, keeping nets away from fire and reducing the frequency of washing. Reach of a national LLIN campaign message was similar between districts, with limited exposure of respondents to this message (18.1 %). The mean number of messages recalled was significantly higher in the intervention district (4.6 messages) than in the comparison district (3.0 messages; P < 0.001) at endline.

Significantly more families in the intervention district (83.6 %) reported discussing net care and repair in the preceding 12 months compared to the comparison district (51.1 %; P < 0.001). Additionally, significantly more children had discussed net care and repair in their families in the past 12 months in the intervention (49.7 %) compared to the comparison district (23.5 %; P < 0.001).

### Knowledge about net care

More people in the intervention district (58.5 %) identified repairing holes as a method to take care of their nets compared to the comparison district (10.3 %; Table 2). In addition, respondents in the intervention district mentioned more often to protect nets from damage inflicted by rodents and children. In contrast, more people in the comparison district mentioned that nets should be washed less often and with ordinary soap (Table 2).

**Table 2:** Percent distribution of knowledge about net care and repair at endline

	Endline		P value^π^
	Comparison N = 445	Intervention N = 425	
How can people take care of their nets?^a^			
Handle nets with care	28.3	39.9	0.059
Keep away from flame or fire	17.6	16.6	0.800
Keep away from rats	11.4	21.3	0.007
Keep away from children	15.5	29.3	0.003
Roll up or tie up when not in use	51.1	57.0	0.215
Wash nets less often	31.9	16.7	0.001
Wash nets with ordinary soap	73.9	62.0	0.014
Dry nets in shade	52.7	61.2	0.080
Repair holes	10.3	58.5	<0.001
What is the recommended way to repair a net?^a^			
Repair holes immediately	6.2	21.8	<0.001
Sew	64.6	79.8	0.007
Tie	29.4	41.9	0.020
Patch	2.2	13.1	<0.001
Do not know	16.5	0.3	<0.001
Composite attitude index			
Poor/average attitude	60.5	37.3	<0.001
Positive attitude	29.5	30.9	
Very positive attitude	10.0	31.8	

^π^P values compare responses between districts for endline only

^a^Answers mentioned <10 % and “Other” not included

Significantly more people in the intervention district were able to cite various methods to repair a net, including sewing, tying and patching. In the comparison district, more often respondents indicated they did not know what the recommended way was to repair a net (16.5 %) compared to only 0.3 % in the intervention district (Table 2). In addition, repairing holes early was significantly more often mentioned in the intervention compared to the comparison district.

**Fig. 3:**
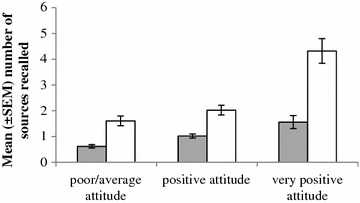
Mean (±SEM) number of sources respondents could recall grouped by their attitude score for comparison (*grey*) and intervention (*white*) districts

The composite attitude index showed that respondents in the intervention had a more positive attitude, with 31.8 % of respondents classified as having a very positive attitude towards nets and net care and repair, compared to 10 % in the comparison district (Table 2). The number of sources respondents could recall regarding messages on net care and repair was positively associated with their attitude in both districts (P < 0.001; Fig. 3). Additionally, respondents that could recall more messages had a better attitude in both districts (P < 0.001).

At baseline there was no significant difference in the average number of times respondents thought a mosquito net should be washed each year in both districts, and the average frequency was 18.4 washes. In the comparison district there was no change at endline. However, in the intervention district a significant reduction was observed at endline compared to baseline figures (P < 0.001) and respondents indicated that a net should be washed on average 9.9 times a year. The majority of people in the intervention district knew that nets should be washed gently (55.5 %), and with ordinary soap, not detergent (73.7 %), and this was significantly higher compared to respondents in the comparison district where 34.8 % of respondents knew that nets should be washed gently and 55.0 % mentioned ordinary soap, not detergent. Respondents in the comparison district, however, overwhelmingly knew that nets should be washed in a basin (82.2 %) compared to respondents from the intervention district (33.0 %). Also, in the comparison district, significantly more people knew that nets should only be washed when dirty.

### Net care and repair behaviours

**Fig. 4:**
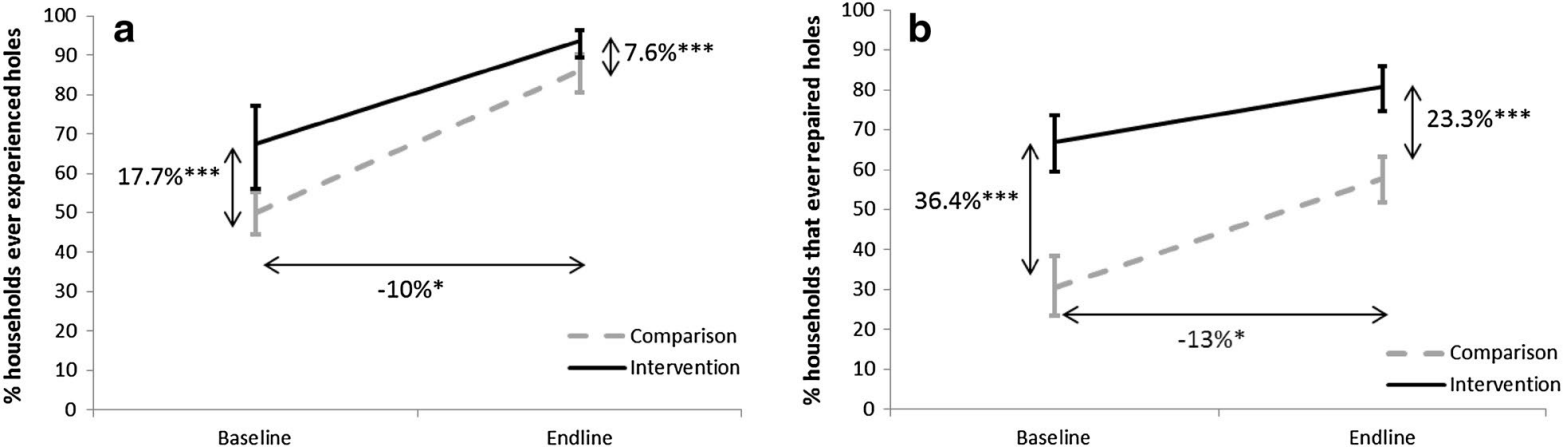
Percent of respondents [95 % CI] that **a** ever experienced holes in their nets or **b** ever repaired holes in their nets, by survey and district. P values for the differences at baseline and endline between districts are indicated. Additionally, the difference in difference is indicated; whereby a *negative DID* indicates that the comparison group had a greater change over time compared to the intervention group; *P < 0.05; ***P < 0.001

At baseline, significantly more respondents in the intervention district reported to have ever experienced holes in any of the nets they owned compared to the comparison district (Fig. 4a). Additionally, significantly more respondents in the intervention district reported to have ever tried to repair these holes (Fig. 4b). Because of these inherent differences in reported behaviour and experience prior to the BCC programme, a difference in difference (DID) was calculated.

The percent of respondents that had noticed holes in their nets increased in both districts, and at endline 86.0 and 93.6 % of respondents had ever experienced holes in their nets in the comparison and intervention districts, respectively, yet the the rate of increase was significantly greater in the comparison district compared to the intervention district (Fig. 4a). Similarly, the percent of respondents that had ever tried to repair holes increased in both districts compared to baseline figures, yet in the comparison district a significantly greater increase in percent points was observed compared to the intervention district (Fig. 4b).

The main cause of tears/holes in both districts were reported to be rodents (i.e., significantly more often mentioned by respondents in the intervention district (61.1 %) than in the comparison district (50.2 %; P = 0.030 at endline) followed by nets getting caught on edges/nails and nets that were pulled and tore on corners.

Of the households who reported repairing nets at endline, the majority of repairs were done using stitching (68.7 and 86.8 % in the comparison and intervention district, respectively), followed by knotting or tying. The majority of repairs were done by the respondents of the survey, which were mainly the heads of households or their spouses. Of the households who did not repair nets at endline, more respondents in the comparison district indicated they did not know how to repair nets (31.4 %) compared to 11.2 % of respondents in the intervention district (P = 0.003). Other common reasons mentioned for not repairing nets were not enough time for repairs, materials not available for repairs, and too difficult to repair.

### Campaign nets distribution and attrition

Households received on average 2.4 nets from the campaign in 2012 (Table 3). During baseline it was observed that the net distribution campaign did not distribute the same nets in both districts. The comparison district had received exclusively Olyset nets while in the intervention district PermaNet was almost exclusively distributed, alongside a few Olyset nets (i.e., 5.5 %). The proportion of all nets that were campaign nets increased in both districts at endline compared to baseline figures, most likely because older, non-campaign nets had been discarded in the meantime.

**Table 3:** Percent distribution of selected characteristics about nets received from the campaign that were lost

	Baseline			Endline		
	Comparison	Intervention	P value^π^	Comparison	Intervention	P value^π^
	N = 428	N = 450		N = 445	N = 425	
Average nets received [95 % CI]	2.5 [2.3–2.7]	2.3 [2.2–2.4]	0.045	2.3 [2.1–2.5]	2.3 [2.2–2.4]	0.991
Proportion of households still with all nets given [95 % CI]	94.5 [92.7–96.2]	95.6 [92.9–98.4]	0.476	82.7 [77.8–87.6]	86.8 [83.3–90.3]	0.177
Average age losing the net, months [95 % CI]	1.8 [1.3–2.3]	1.6 [1.2–2.0]	0.593	10.3 [8.9–11.7]	6.6 [5.3–7.9]	<0.001
Attrition overall [95 % CI]	2.3 [1.3–3.3]	2.2 [0.8–3.6]	0.869	8.9 [6.4–11.4]	6.8 [4.9–8.7]	0.190
Attrition due to wear and tear [95 % CI]^a^	0.6 [0–1.2]	0.2 [0–0.4]	0.149	5.4 [3.6–7.3]	1.7 [0.9–2.4]	<0.001

^π^P values are shown for baseline and endline comparisons separately

^a^Only including nets destroyed, nets lost to wear and tear, other and do not know answers

The percent of households that still had all campaign nets given to them was 82.7 and 86.8 % at endline in the comparison and intervention district, respectively (Table 3). Overall attrition was 8.9 % of nets in the comparison district, and 6.8 % of nets in the intervention district (Table 3) at endline. When only taking into account those nets lost due to wear and tear, attrition was significantly higher in the comparison district, with an attrition rate of 5.4 % compared to 1.7 % in the intervention district.

Almost half of all nets in the intervention district that were no longer in the possession of the household were reported to be lost within the first 3 months after receiving the nets (48.5 %) while in the comparison district the majority of nets were lost within 6–12 months after receiving them (44.9 %). The average age that nets were lost in the intervention district was thus lower than in the comparison district (Table 3). At baseline, the large majority of nets no longer in the possession of the household were given away to relatives or others (80.8 %), indicating that these nets were not lost but rather redistributed. At endline this was still the case for the intervention district and 62.6 % of all lost nets had been given away to others, against only 35.4 % in the comparison district. In the comparison district, a much larger number of respondents indicated having thrown away the net (49.0 %) compared to the intervention district (17.1 %; P = 0.036). The main reason for no longer keeping the net in the comparison district was because it was too torn (53.7 %), while in the intervention district most often respondents indicated that they had given the net away to others because they needed it more (45.3 %; P = 0.007).

### Campaign nets, location and use

During the endline survey, the majority of campaign nets in the intervention district were observed to hang loose over the sleeping place (61.4 %), compared to 49.3 % in the comparison district (P < 0.001). Although the tying/rolling up of hanging nets was a key message of the BCC intervention to prevent net damage, a similar percent of nets were observed tied in the intervention (26.7 %) compared to the comparison district (27.0 %).

Of all the nets that were reported to be in use (i.e., excluding nets never used), more than 94 % of nets at endline were slept under by a person the previous night, and no differences were observed between intervention and comparison district (P = 0.331). The majority of nets (>91.2 %) were used every night of the week, although use frequency rates were slightly higher in the intervention district during both surveys (P < 0.01). The main reason mentioned for not sleeping under a net was that there were already sufficient nets available in the household and the net was surplus.

### Campaign nets, net care and repair behaviours

Almost all campaign nets had been washed at least once in both districts at endline (>97 %). The average wash frequency was similar between districts and was three washes in the last 3 months, or once a month (P = 0.212). In both districts bar/liquid soap was used by the large majority of respondents for net washing (75.7 % in comparison and 90 % in intervention district). Importantly, bleach was never reported to be used to wash the nets. An increasing number of nets were reported to be dried in the shade as is appropriate in both districts compared to baseline figures, yet significantly more respondents indicated doing this in the intervention district (78.4 %) than in the comparison district (65.8 %; P < 0.001).

### Net durability and survival

Net care and repair behaviour was assessed for the campaign nets that were in use. Analyses were done separately by net brand as there were significant differences in the durability of the two brands observed (P < 0.001). Since no PermaNets were observed in the comparison district, only data for the intervention district are presented for this brand.

At baseline, nets had just been received and few nets were reported to have had holes, and there were no differences between districts in the proportion of nets with any repairs or in net condition (Table 4). Twenty months post-distribution, the large majority of nets had at least one hole. More Olyset nets in the intervention district were reported to have had a hole compared to nets in the comparison district. The percent of Olyset nets with evidence of any repair done (i.e., full or partial) was similar between districts and varied between 48.0 % of nets for the comparison district and 56.4 % of nets for the intervention district. The average number of repairs done, either full or partial, per net were greater in the intervention than in the comparison district (Table 4). Net condition was worse in the intervention district, where 20–21 months after net distribution on average 42.2 % of Olyset nets in the intervention district had survived in ‘serviceable’ condition, against 55.4 % of nets in the comparison district. The PermaNet brand nets were in better condition, and 74.6 % of nets were considered serviceable (Table 4).

**Table 4:** Percent distribution of level wear and tear (holes) and repair practices of the nets received from the campaign

	Baseline			Endline			
				Olyset sample			PermaNet sample
	Comparison	Intervention	P value^π^	Comparison	Intervention	P value^π^	Intervention
Net ever had a hole	N = 795	N = 695		N = 718	N = 535		N = 718
Yes [95 % CI]	11.1 [8.3–13.9]	15.4 [7.2–23.6]	0.329	82.5 [76.5–88.4]	93.0 [88.3–97.7]	0.008	86.5 [81.9–91.0]
Hole repairs [95 % CI]	N = 90	N = 98		N = 604	N = 497		N = 619
Any repairs	3.4	5.6	0.559	48.0	56.4	0.179	60.1
No. of full repairs, mean [95 % CI]	0.0	0.1 [0.0–0.2]	0.098	0.6 [0.5–0.8]	1.8 [1.4–2.2]	<0.001	1.3 [1.1–1.6]
No. of partial repairs, mean [95 % CI]	0.0 [0.0–0.1]	0.1 [0.0–0.2)	0.635	0.7 [0.5–0.8]	1.5 [0.9–2.2]	0.015	1.5 [1.2–1.9]
Proportionate hole index (pHI), median^a^	N = 90	N = 98		N = 718	N = 535		N = 718
Overall	26	60	0.154	830	837	0.045	275
Net condition (based on pHI category)							
Good (pHI < 64)	96.0	92.4	0.231	32.6	14.2	0.017	38.1
Damaged (pHI 65–642)	3.1	5.0		22.8	28.0		36.5
Too torn (pHI > 642)	0.8	2.5		44.7	57.8		25.4
Serviceable (pHI 0–642)	99.2	97.5	0.196	55.4	42.2	0.034	74.6
Too torn (pHI > 642)	0.8	2.5		44.7	57.8		25.4

The endline results are presented for nets identified as Olyset in comparison and intervention district and nets identified as PermaNet for the intervention district only

Nets still in package were excluded

^π^P values are shown for each comparison between districts separately

^a^Nets without holes were excluded

Net survival, i.e., the proportion of received nets not given away for use by others that are still present and in “serviceable” condition for Olyset nets overall was estimated at 55.2 % [95 % CI 48.2–62.3] which was similar to a two-year median survival curve, while for the PermaNet sample survival was 74.3 % [95 % CI 68.3–80.4] and nets followed closer to a 3 year median survival curve.

### Predictors of net condition

**Fig. 5:**
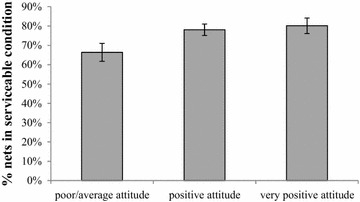
Percent of PermaNets in serviceable condition by attitude. Results are for endline survey and for intervention district only

A number of factors were assessed to see if they could predict net outcome using a logistic regression model. The main analysis was done for PermaNets as these nets were in significantly better overall condition. Exposure to net care and repair messages, respondent’s attitude towards net care and repair, and socioeconomic status were all positively associated with a better net condition (Table 5). Seventy-eight and 80 % of PermaNets belonging to respondents with a positive or very positive attitude, respectively were in serviceable condition, compared to only 66 % of nets from respondents classified as having a poor to average attitude (Fig. 5). Increasing washing frequency was negatively associated with net condition. In addition, nets that had any signs of repair were in significantly poorer condition compared to nets that had not been repaired. Some other predictors, i.e., the presence of children under the age of five in the household, the number of messages recalled, sleeping material used, universal coverage status of the household, and reported presence of rodents did not have a significant impact on net condition. After adjusting for other variables, SES, repair status of the net and attitude were still significant in the final adjusted model for PermaNet (Table 5), with SES and attitude positively associated with net condition, while net repairs were negatively associated with net condition.

**Table 5:** Odds ratios (OR) with their 95 % confidence intervals and P values for predictors used in the logistic regression model for net condition (1: serviceable, 0: too torn) for the PermaNet brand

Predictors	Crude		Adjusted	
	OR (CI)	P value	OR (CI)	P value
Heard any messages net care and repair (*ref: no*)				
Yes	1.50 [1.03–2.19]	0.036		
Signs of repair (*ref: no*)				
Yes	0.33 [0.24–0.47]	<0.001	0.33 [0.22–0.49]	<0.001
Children under 5 years (*ref: no*)				
Yes	0.81 [0.46–1.42]	0.446		
Sleeping material (*ref: Ground/reed mat*)				
Foam mattress	1.84 [0.98–3.44]	0.168		
Frame finished	1.72 [0.89–3.34]			
LLIN hanging & tied up (*ref: no*)				
Yes	1.19 [0.73–1.95]	0.468		
Attitude (*ref: poor/average*)				
Positive	1.80 [1.10–2.96]	0.028	1.77 [1.03–3.05]	0.040
Very positive	2.05 [1.07–3.94]		2.11 [1.01–2.88]	0.046
Net washing frequency (*ref:* ≤*1 times*)				
2–3 times	0.93 [0.62–1.40]	0.017		
>3 times	0.58 [0.40–0.85]			
Household universally covered (*ref: no*)				
Yes	1.29 [0.87–1.92]	0.189		
Presence of rats (*ref: no*)				
Yes	0.63 [0.30–1.32]	0.210		
Social economic status (*ref: lowest*)				
Second	2.08 [1.01–4.28]	<0.001	2.70 [1.23–5.94]	0.015
Third	2.38 [1.28–4.42]		2.61 [1.27–4.97]	0.010
Fourth	3.68 [1.65–8.19]		3.11 [1.60–6.03]	0.002
Highest	5.19 [2.69–9.99]		4.16 [2.11–8.18]	<0.001

The crude predictors are presented, as well as the final adjusted model

When a multivariate model was run for Olyset nets, net repairs were also more commonly seen for nets in ‘too torn’ condition (P < 0.001). Additionally, presence of rodents was negatively associated with net condition (P = 0.005), and nets that were used over reed mats or sleeping spaces on the ground were significantly more often classified as ‘too torn’ than nets used over bed frames or foam mattresses (P < 0.003), while nets that were hanging folded or tied up over a sleeping space were less often classified as ‘too torn’ compared to nets that were not (P = 0.019). SES, attitude and washing frequency were not significant. In this model, net condition was no longer significantly different between districts (P = 0.060).

## Discussion

The main objective of the study was to assess whether the BCC programme could positively impact on net durability. While the BCC programme was successful in reaching a large proportion of households in the intervention district, results show that durability of Olyset nets was similar in the intervention and comparison areas at endline. However, the uneven distribution of net brands and overall poor durability of Olyset nets make it difficult to draw conclusions on whether the lack of impact observed was due wholly to failure of the BCC programme. Further analysis within the intervention district (PermaNets) showed that the number of channels heard and number of messages recalled were positively associated with respondents’ attitude towards net care and repair, and that, in turn, nets belonging to respondents with a more positive attitude were in significantly better condition. However, repairs made to nets did not improve their condition. The findings indicate that BCC may play a role in improved net condition by improving attitudes towards care and repair.

The BCC intervention was a multi-channel activity that used mass media messaging, interpersonal communication, and school and community events as its main channels. Respondents in the intervention area reported a significantly increased exposure to net care and repair messages compared to baseline levels, but this trend was also observed to a lesser, but still significant degree in the comparison district. This is likely due to mass media of the ongoing national LLIN distribution campaign that was taking place countrywide. Indeed, at least one message from the national campaign was remembered by around one-fifth of respondents in the intervention and comparison districts, indicating a similar reach in both districts. In addition, some messaging from the BCC intervention in the intervention district may have reached the comparison district. During the design phase, intervention district representatives specifically requested that activities take place in Teso and English as they were the dominant languages in their district. The addition of English may have made it easier for messages to reach the comparison district, although a specific slogan from the BCC intervention was only recalled by 1 % of respondents in the comparison district.

The BCC campaign stressed the importance of handling nets with care, only washing nets when dirty, and the benefits these actions would give (less malaria, better sleep, saving money). In addition, substantial emphasis was placed on the inspection of nets and early repair of holes. The intervention led to an overall improved knowledge of respondents in the intervention district. In addition, the attitude of respondents towards net care and repair was better in the intervention district, although no baseline levels were available. In both districts, but to a greater degree in the intervention area, a positive relationship between the dose, i.e., number of channels respondents had been exposed to, and attitude was observed. This dose–response relationship, as observed in a similar study in Nigeria [18], provides an indication that a plausible causal relationship between exposure and attitudes is present.

Better attitudes, in turn, were positively associated with more PermaNets being in serviceable condition at endline in the intervention district when controlling for other factors, and a similar positive effect of attitude on net condition was found in a similar study performed in Nigeria [18]. The same was not observed for the Olyset nets, which were in general in poor condition in both districts, and their lifespan, with around half of all surveyed nets found in too torn condition approximately 2 years post-distribution, followed a two-year net survival curve [21]. Poor durability of Olyset nets was observed in other studies [7, 22–24], and the manufacturer has since changed the knitting pattern of the product (Nick Brown, personal communication). Given that the intervention-area Olysets showed no improvement over Olysets in the comparison district, it seems likely that while behaviour change interventions can improve net care and repair behaviours and improve net condition, there is a limit in its ability to prevent deterioration when the physical construction of LLINs is suboptimal.

Repair was a large focus in the BCC intervention as it was hypothesized to have a significant impact on the pHI. The proportion of nets with observed repairs was similar between districts, with approximately 50 % of all nets showing signs of repair, although more repairs per net were achieved in the intervention district. However, this increased repair behaviour did not contribute to better net condition, and when including other predictors in the model, there was no significant difference in the percent of nets classified as serviceable for the Olyset brand between both districts. In fact, repaired nets were in significantly worse condition than non-repaired nets for all brands assessed, which was also observed in the Nigeria study [18]. This suggests that repairs were not made early or thoroughly enough to move a net into a less-damaged pHI category. The findings reported here mirror those in the Gambia, where an increase in net repairs following a BCC intervention was also observed. However, nets remained badly torn and very few households succeeded in repairing all of the holes in their net [17]. It is possible that repairs may have been initiated too late, once the net condition had already deteriorated so significantly that it was beyond repair, and additional studies are required to understand whether early repairs can make a significant impact. On the other hand, attitudes towards repair specifically were an integral component of the overall attitude score that significantly contributed to improved net condition. It is reasonable from this data that repair messages influence overall attitudes, and that therefore, while repair behaviour is not sufficient to improve net condition, repair messages still play an important role in establishing and strengthening the attitudes that improve the standing of a net in a household to a highly valued item that should be well-cared for. Qualitative data collected from the area and from another study in Nigeria identified a number of motivators for net care and repair, including caring for one’s family, avoiding mosquito bites, saving money, and maintaining the positive opinion of others by keeping a clean and intact net [4, 19]. Repair messages should, therefore, not be ignored when designing BCC activities to improve net integrity.

Looking at the PermaNets in the study, the other factors that impacted on net condition included socioeconomic status of the household, with wealthier households having nets in better condition. More frequent washing (more than three times in the previous 3 months) was associated with poorer net condition. For Olyset nets it was observed that nets that were used over reed mats or for sleeping places located on the ground were less likely to be in serviceable condition than nets observed over foam mattresses or bed frames, likely due to the fabric integrity issues of this group of nets. In addition, presence of rodents negatively impacted on Olyset condition. Presence of rodents was negatively associated with net condition in some parts of Nigeria [25]. The different predictors that were observed for each net brand suggest that factors which may contribute towards a serviceable or too torn net condition are dependent on physical integrity; weaker nets may be more susceptible to some factors earlier in their lifespan compared to stronger nets. A similar study in Nigeria also observed that 100 denier polyester nets were more likely to be in serviceable condition in wealthier quintiles, and if the net was tied up during the day, but that neither washing frequency, presence of rodents, nor education level of the head of household had a significant effect on net condition, in a multivariate regression model [18]. While some of the predictors observed to influence net condition may be more easily addressed by household owners themselves, such as washing frequencies, households are less able to reduce their rodent populations, or easily improve their bedding materials. This is particularly the case for the poorest quintile, whose nets were more likely to be too torn than were the higher quintiles. Understanding the behavioural actions that people with a more positive attitude towards net care and repair take should be investigated further.

Net use was overall high in this survey. Although net coverage of at least one net for every household remained high, the majority of households no longer had enough nets to cover all of its residents 20–21 months post-distribution. Although the two districts, alongside the other two that received nets in September 2012, did not receive any additional nets from the LLIN mass campaign in 2013/2014, results suggest that especially districts that received Olyset nets require replacing nets in the immediate future through either campaigns or continuous distribution to achieve high levels of net ownership.

Although the use of this multiple-channel BCC intervention was beneficial in improving attitudes around net care and repair, more research about the combination of channels that is most significant would be useful. Such research should also segment participants by vulnerability to net damage. Unlike many HIV and family planning messages, net care is not sensitive and can be openly discussed in large fora. For this reason, care messages may be easily added to radio and news programmes and to large community events and achieve high reach. However, the poorest households—who are also more likely to have nets in poor condition, as well as less educated, less literate, less likely to access services and also sleeping on mats—are less likely to be reached by radio, and may benefit from targeted interpersonal interventions. Community health workers, who are already tasked with identifying and reaching the poorest households, can include net care messages in their work. Instead of having a stand-alone campaign, programme planners may be able to leverage existing programmes. Net care messages can be included as part of net use messaging in other BCC campaigns, community case management programmes and in continuous net distribution programmes where nets are distributed by health workers, school teachers or community-based individuals. Other programmes may also be able to convince education officials to incorporate net care and repair into school curricula. Although this study evaluated a BCC intervention (specifically, a combination of mass media and community/school activities), other approaches to bring about change in behaviour, such as community-directed approaches, core groups/support groups or positive deviance, may also be effective in improving net care behaviour. Introducing net care messages may in fact, help make net use messages feel “fresh” to communities with years of exposure to net use messaging.

### Strengths and limitations

This is one of few studies documenting the impact of a BCC intervention on household behaviour and net condition over time. The study was hampered by the unanticipated uneven distribution of net brands in the intervention and control districts, and the overall poor durability of Olyset nets, which complicated the analyses and interpretation of study findings. Different net brands were used because the nets for the campaign distribution in those districts did not arrive in time. As a result, nets were pulled from the stock that was in country at the time.

There is some indication that the success of the BCC intervention was limited by its late start, 9 months after the nets had been distributed. Given the rate of decay it is likely that nets had already acquired holes and some might have already been beyond repair during these initial months. Indeed, nets in the comparison district had been predominantly lost from household 6–12 months post-distribution, and the main reason indicated was that net was too old/torn.

This study rested on the premise that a net in serviceable condition is more protective against malaria. More research on the relationship between pHI and malaria prevention is needed to fully understand the contribution of net care and repair behaviours to malaria prevention.

## Conclusions

The evaluation showed that the BCC programme resulted in improved knowledge and attitudes of respondents, which impacted positively on net condition. This was likely the result of overall better care for the nets. Repairs themselves were not sufficient to improve net condition, although repair attitudes were a critical component of the attitudes that positively affected net condition. Additional research is needed to understand whether early repairs could improve net condition. As such, BCC messages on net care and repair should be part of malaria control programmes and can be integrated in existing services. This study also provided specific recommendations for such programmes as it lent insights on channel, audience and message selection. Due to the range of care behaviours that can be promoted, additional research on which attitudes and care behaviours are most associated with improved net condition would help BCC planners hone their campaigns.
